# Case–Controlled Clinical Assessment of the Olfactory System via Cranial Magnetic Resonance Imaging in Patients With Type 2 Diabetes Mellitus

**DOI:** 10.1002/brb3.71210

**Published:** 2026-01-15

**Authors:** Aski Vural, Erman Altunişik, Suat Kamil Sut, Sukru Sahin, Ali Haydar Baykan

**Affiliations:** ^1^ Department of Internal Medicine, Faculty of Medicine Adiyaman University Adiyaman Turkey; ^2^ Department of Neurology, Gaziantep City Health Application and Research Center Health Sciences University Gaziantep Turkey; ^3^ Department of Radiology Sincan Training and Research Hospital Ankara Turkey; ^4^ Department of Radiology Elazığ Fethi Sekin Training and Research Hospital Elazığ Turkey; ^5^ Department of Radiology, Faculty of Medicine Adiyaman University Adiyaman Turkey

**Keywords:** diabetes mellitus, magnetic resonance imaging, olfactory bulb volume, olfactory sulcus depth, olfactory tract length

## Abstract

**Objective:**

The purpose of this study is to assess the olfactory system of patients with T2DM using the cranial magnetic resonance imaging (MRI) method.

**Method:**

This is a retrospective case–control study in which a group of T2DM patients and a control group were compared. The results of the examinations of the olfactory systems of the patients by cranial MRI were transferred to a data collection form. Descriptive statistical methods, chi‐squared tests, the Mann–Whitney *U* test, and Spearman's correlation coefficient were used to analyze the data.

**Results:**

It was determined that 66.7% of the case group were women, the mean age of the patients in the group was 52.50 ± 7.41, and their mean T2DM diagnosis duration was 6.48 ± 3.18 years. There were statistically significant differences between the case and control groups in terms of their olfactory bulb volume (OBV), olfactory tract length (OTL), and olfactory sulcus depth (OS) values. Longer T2DM durations and elevated HbA1c levels were significantly associated with structural disorders of the olfactory system (*p* < 0.01).

**Conclusion:**

A longer duration of T2DM and elevated HbA1c levels trigger the structural disorders of the olfactory system. In comparison to healthy controls, we identified prominent changes in the olfactory bulb volumes, olfactory tract lengths, and olfactory sulcus depths of T2DM patients. This reveals the need for T2DM patients to pay more attention to their diet and insulin treatment. Similarly, olfactory dysfunction in T2DM patients should be carefully monitored by clinicians.

## Graphical abstract

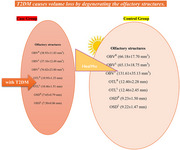

## 1 Introduction

In 2019, 463 million patients with diabetes mellitus (DM) were reported worldwide, while it is expected that this number will reach about 700 million by a 51% increase in 2045. The number of DM patients in Turkey was 3.679 million (prevalence 8%) in 2010, which later increased to 6.592 million (prevalence 11.1%) in 2019 (International Diabetes Federation 2019). The prevalence of total Type 2 DM (T2DM) was reported as 15.8%, diagnosed diabetes was 11.3%, and undiagnosed diabetes was 4.5% in the United States (Gwira et al. 2024). More than 90% of patients diagnosed with DM have T2DM (Stumvoll et al. 2005; Weyer et al. 1999). T2DM develops as a result of the insufficient secretion of insulin by pancreatic β‐cells or a lack of response to insulin in sensitive cells and insulin resistance (Roden and Shulman 2019).

As T2DM progresses, the balance between insulin and glucose is disrupted, and hyperglycemia develops. β‐Cell dysfunction may originate from different molecular pathways in cell physiology and changes in signaling on a complicated transmission network between structures of neighboring cells (Halban et al. 2014). Besides, uncontrolled β‐cell death may also trigger T2DM (Christensen and Gannon 2019). In addition to the pancreas, the development of T2DM may be influenced by the kidneys, liver, small intestine, brain, and adipose and muscle tissue (Defronzo 2009). Brain tissue affected by T2DM is damaged, and the resulting neurodegeneration leads to deterioration in olfactory system structures (Reyes 2025; Sanke et al. 2014; Walliczek‐Dworschak et al. 2017). Nevertheless, upper respiratory tract infections are the most frequently encountered causes of olfactory dysfunction. Other causes include psychiatric diseases, alcoholism, exposure to chemical substances, and intracranial tumors (Brady et al. 2013; Zaghloul et al. 2018). Considering all these problems, in the literature, the prevalence of olfactory dysfunction was reported to vary from 2.7% to 76.8% in individuals (Doty 2019). Moreover, it is thought that 22% of patients with T2DM have olfactory dysfunction (Rasmussen et al. 2018).

Olfactory dysfunction may be caused by the effect of a chemical/biological agent or a small cytokine storm emerging in the mucosa and olfactory nerve due to inflammation (Ralli et al. 2020). Olfactory nerve dysfunction may develop as a result of chemosensory involvement, nasal obstruction, and excessive dehydration (Nehlig 2016). Considering the pathophysiology of the disease in individuals with T2DM, olfactory dysfunction can be associated with olfactory nerve dysfunction and chemosensory involvement. In this sense, smell disorders developing due to olfactory dysfunction may be a predictor of neurodegeneration and nerve damage that can be associated with further clinical deterioration. In this study, we aimed to examine the olfactory system structures of patients with T2DM via magnetic resonance imaging (MRI).

## 2 Materials and Methods

We conducted this study with a case–control design to investigate the olfactory structures of patients receiving treatment for the diagnosis of T2DM using the MRI method.

### 2.1 Design and Participants

The data collection process in this case–control study was carried out retrospectively. Individuals aged 18–60 who were being followed up at the internal medicine and neurology clinics of a hospital in eastern Turkey constituted the sample. Patients with T2DM were included in the case group, whereas others were included in the control group. The control group included 18‐ to 60‐year‐old male and female patients who had undergone cranial MRI scans due to another nondegenerative reason (e.g., headache, dizziness, and tinnitus) and were not diagnosed with DM. Data were retrospectively obtained from electronic patient records. The sample size required to conduct the study with a 0.05 margin of error and 95% confidence interval was found to be 74 in the power analysis conducted using the G*Power‐3.1.9.2 software. The case group included 38 patients with T2DM, whereas the control group included 80 healthy individuals who were not diabetic. The inclusion criteria used to select participants for the case and control groups are presented below.

### 2.2 Inclusion Criteria

The inclusion criteria for the case group were as follows: (i) having been diagnosed with T2DM at least 1 year ago, (ii) being a non‐smoker who does not consume alcohol, and (iii) not having a comorbid disease other than T2DM. The inclusion criteria for the control group were as follows: (i) not having any comorbid disease, (ii) being a non‐smoker who does not consume alcohol, and (iii) having no complaints of olfactory dysfunction. Both groups included individuals aged 18–60.

### 2.3 Cranial MRI Protocol

The patients in the case and control groups underwent cranial (MRI) scans using a 1.5‐Tesla MRI protocol (1.5‐Tesla MR scanner) (Signa Explorer 1.5 Tesla MRI imaging machine manufactured by GE Healthcare, employing a 16‐channel head coil). Previous studies have emphasized that MRI is a valid and reliable method to examine olfactory system structures (Glaser et al. 2006; Gil‐Carcedo et al. 2000; Han et al. 2019; Mueller et al. 2005). In the MRI scans, the coronal cross‐section was imaged in the fast imaging sequence employing steady‐state acquisition with phase cycling (FIESTA‐C) (repetition time [TR] of 7.6 ms, echo time [TE] of 2.7 ms, field of view [FOV] measuring 170 × 170 mm, a total of four excitations, slice thickness of 1.6 mm with no interslice gap, 1024 sections, and a matrix size of 300 × 300 mm).

### 2.4 Image Examination

**FIGURE 1:**
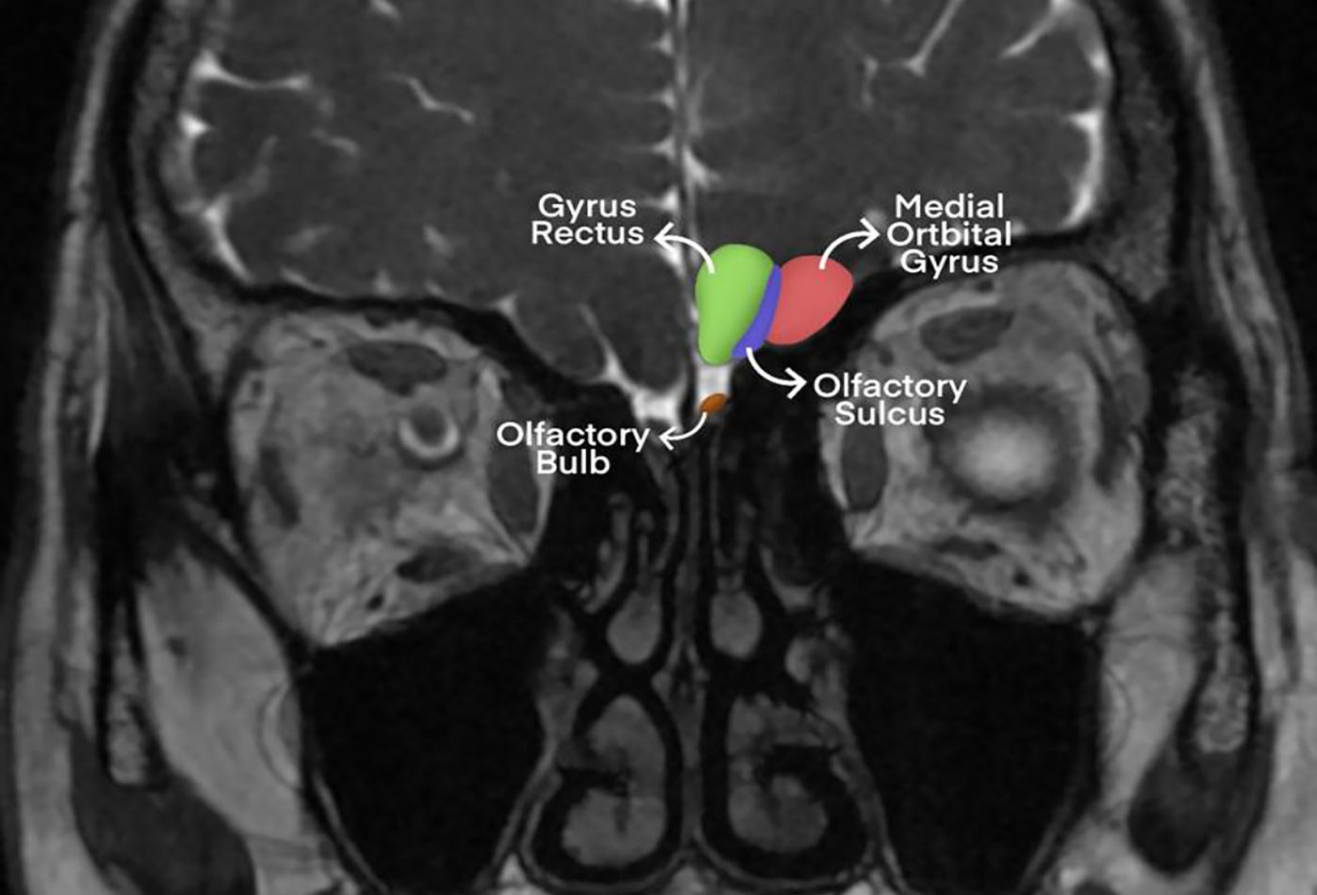
**Olfactory system structures**. Coronal plane MR image of the olfactory system: medial orbital gyrus (red), gyrus rectus (green), olfactory sulcus (blue), and olfactory bulb (brown).

The boundaries of the olfactory bulb (OB) were identified by checking the hyperintensities of the cerebrospinal fluid (CSF) around OB (Chen et al. 2014; Rombaux et al. 2009; Wang et al. 2011; Yousem et al. 1998). Proximal boundaries were identified based on the cribriform plate. Due to the possibility of differences between the right and left OB volumes (OBV) in humans (Wang et al. 2011), both sides were measured (Figure 1). Contouring around OB was performed by utilizing CSF on the coronal plane in the FIESTA‐C sequence, and OB boundaries were manually drawn. Volumes were measured through manual segmentation with the contour stack principle on a three‐dimensional workstation, and OBV values are expressed in cubic millimeters. Olfactory tract length (OTL) values were obtained in millimeters using multiplanar reconstructions of FIESTA‐C 3D sagittal images at the section providing the optimal visualization of the entire nerve tract. Olfactory sulcus depth (OSD) was measured on coronal 3D FIESTA‐C images by drawing a line tangential to the inferior boundaries of the gyrus rectus and medial orbital gyrus, quantifying the deepest point between these gyri to obtain values in millimeters. Two radiologists, each with at least 10 years of experience in head and neck radiology, performed all volume and length analyses, and the measurements of both radiologists were averaged to increase reliability.

### 2.5 Statistical Analysis

The SPSS 27.0 statistical analysis package program was used to analyze the collected data. The Kolmogorov–Smirnov *Z*‐test was conducted to determine whether the data were normally distributed. Descriptive statistics were calculated as frequency, percentage, mean, and standard deviation values. The chi‐squared test was conducted to determine whether there was a significant difference between the sex distributions of the case and control groups. The Mann–Whitney *U* test was carried out to determine whether the ages of the patients differed significantly between the case and control groups. The comparisons of OB, OTL, and OSD values between the case and control groups were also made using the Mann–Whitney *U* test. Spearman's correlation coefficient was used to determine the relationships between DM duration and HbA1c levels and the right OBV, left OBV, total OBV, right OTL, left OTL, right OSD, and left OSD values. In the analyses, *p* < 0.05 was selected as the threshold for statistical significance.

### 2.6 Ethical Aspect of the Research

Before this research, an Institutional Review Board was obtained from the Department of Internal Medicine, Adiyaman University, Faculty of Medicine, Training and Research Hospital. Ethics Committee permission was obtained from the Adiyaman University Clinical Research Ethics Committee (Date: 15 Jun 2021, Number: 2021/06‐12). The research steps were followed in accordance with the Helsinki Declaration. Written and verbal consent was obtained for the use of patients MRI images regarding the study.

## 3 Results

Table 1 presents the individual characteristics and homogeneity test results of the case and control groups. As seen in Table 1, 66.7% of the patients in the case group were women. The mean age of the patients in the case group was 52.50 ± 7.41, and their mean duration of T2DM diagnosis was 6.48 ± 3.18 years. Moreover, 71.3% of the patients in the control group were women, and the mean age of the patients in the group was 50.43 ± 9.12. There was no significant difference between the case and control groups in terms of their sex or age.

**TABLE 1:** Individual characteristics and homogeneity test results of case and control groups (*N* = 118).

Characteristics			
Sex	Case *n* (%)	Control *n* (%)	Test and sig.
Male	14 (33.3)	23 (28.7)	*χ* ^2^ = 1185 *p* = 0.209
Female	24 (66.7)	57 (71.3)	
	x̄ ± SD	x̄ ± SD	
Age (Min, max)	52.50 ± 7.41 (25, 60)	50.43 ± 9.12 (25, 60)	*U* = 0.906 *p* = 0.162
T2DM duration (years)	6.48 ± 3.18	—	—

*Note*: x̄, mean; SD, standard deviation; *χ^2^*, chi‐squared test; *U*, Mann–Whitney *U* test.

Table 2 shows the OBV, OTL, and OSD values of the case and control groups. Accordingly, there was a significant difference between the case and control groups in terms of their OBV, OTL, and OSD values (*p* < 0.01).

**TABLE 2:** OBV, OTL, and OSD values of case and control groups.

Olfactory structures (measurement)	Case	Control	Test and sig.
	x̄ ± SD	x̄ ± SD	
OBV^R^ (mm^3^)	38.93 ± 11.83	66.18 ± 17.70	*U* = 8.967 *p* = 0.001*
OBV^L^ (mm^3^)	37.10 ± 12.49	65.13 ± 18.75	*U* = 8.815 *p* = 0.001*
OBV^T^ (mm^3^)	76.02 ± 23.80	131.61 ± 35.13	*U* = 9.197 *p* = 0.001*
OTL^R^ (mm)	10.95 ± 1.33	12.40 ± 2.28	*U* = 3.792 *p* < 0.001*
OTL^L^ (mm)	10.46 ± 1.51	12.46 ± 2.45	*U* = 4.820 *p* = 0.003*
OSD^R^ (mm)	7.65 ± 0.79	9.23 ± 1.50	*U* = 6.392 *p* = 0.001*
OSD^L^ (mm)	7.50 ± 0.86	9.22 ± 1.47	*U* = 6.974 *p* = 0.001*

*Note: U*, Mann–Whitney *U* test; x̄, mean; SD, standard deviation.

Abbreviations: L, left; OBV, olfactory bulb volume; OTL, olfactory tract length; OSD, olfactory sulcus depth; R, right; T, total.

*p < 0.01.

**FIGURE 2:**
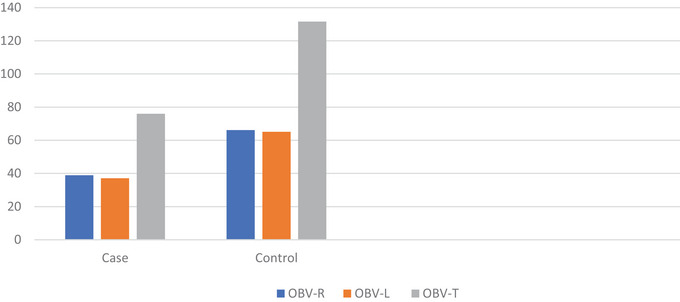
**OBV values of case and control groups**. The right, left, and total OBV values of the control group were almost twice those of the case group.

Figure 2 displays the OBV values of the case and control groups. The mean right OBV values of the patients were 38.93 ± 11.83 in the case group and 66.18 ± 17.70 in the control group. The mean left OBV values were 37.10 ± 12.49 in the case group and 65.13 ± 18.75 in the control group. The mean total OBV values were 76.02 ± 23.80 in the case group and 131.61 ± 35.13 in the control group.

**FIGURE 3:**
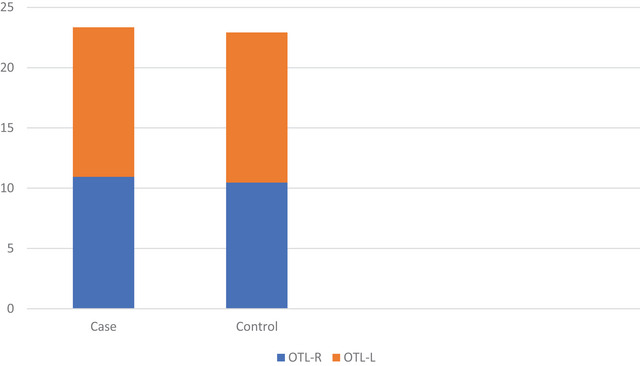
**OTL values of case and control groups**. The control group had higher right and left OTL values than the case group.

Figure 3 displays the OTL values of the case and control groups. The mean right OTL values of the patients were 10.95 ± 1.33 in the case group and 12.40 ± 2.28 in the control group. The mean OTL left values were 10.46 ± 1.51 in the case group and 12.46 ± 2.45 in the control group.

**FIGURE 4:**
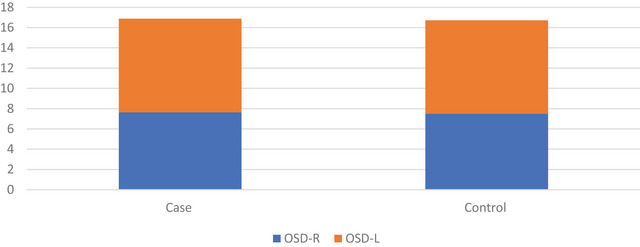
**OSD values of case and control groups**. The control group had higher right and left OSD values than the case group.

Figure 4 displays the OSD values of the case and control groups. The mean right OSD values of the patients were 7.65 ± 0.79 in the case group and 9.23 ± 1.50 in the control group. The mean left OSD values were 7.50 ± 0.86 in the case group and 9.22 ± 1.47 in the control group.

Table 3 shows the results of the correlation analyses between the T2DM durations and HbA1c levels of the case group and their OBV, OTL, and OSD values. The T2DM durations of the patients in the case group were negatively, moderately, and significantly correlated with their OBV^R^, OBV^L^, OBV^T^, OTL^R^, and OTL^L^ values (*p* < 0.05). The T2DM durations of the patients in the case group were not correlated with their OSD^R^ (*p* = 0.082). The HbA1c levels of the patients in the case group were not correlated with their OBV^R^, OBV^L^, and OBV^T^ values (*p* > 0.05). The HbA1c levels of the patients were not correlated with their OTL^L^ and OSD^L^ values (*p* > 0.05). Finally, the HbA1c levels of the patients were positively, significantly, moderately, and weakly correlated with their OTL^R^ and OSD^R^ values (*p* = 0.001).

**TABLE 3:** Correlation analysis between T2DM durations and HbA1c levels of case group and their OBV, OTL, and OSD values.

Olfactory structures	T2DM duration		HbA1c level	
	*r*	*p*	*r*	*p*
OBV^R^	−0.450	0.003**	−0.030	0.850
OBV^L^	−0.406	0.008**	−0.101	0.524
OBV^T^	−0.449	0.003**	−0.068	0.669
OTL^R^	−0.311	0.045*	0.476	0.001**
OTL^L^	−0.571	0.001**	0.195	0.216
OSD^R^	−0.272	0.082	0.206	0.001**
OSD^L^	−0.352	0.022**	0.111	0.216

*Note*: *r*, Spearman's correlation coefficient.

Abbreviations: L, left; OBV, olfactory bulb volume; OTL, olfactory tract length; OSD, olfactory sulcus depth; R, right.

Correlation is significant at the *0.05 level (two‐tailed) and **0.01 level (two‐tailed).

**FIGURE 5:**
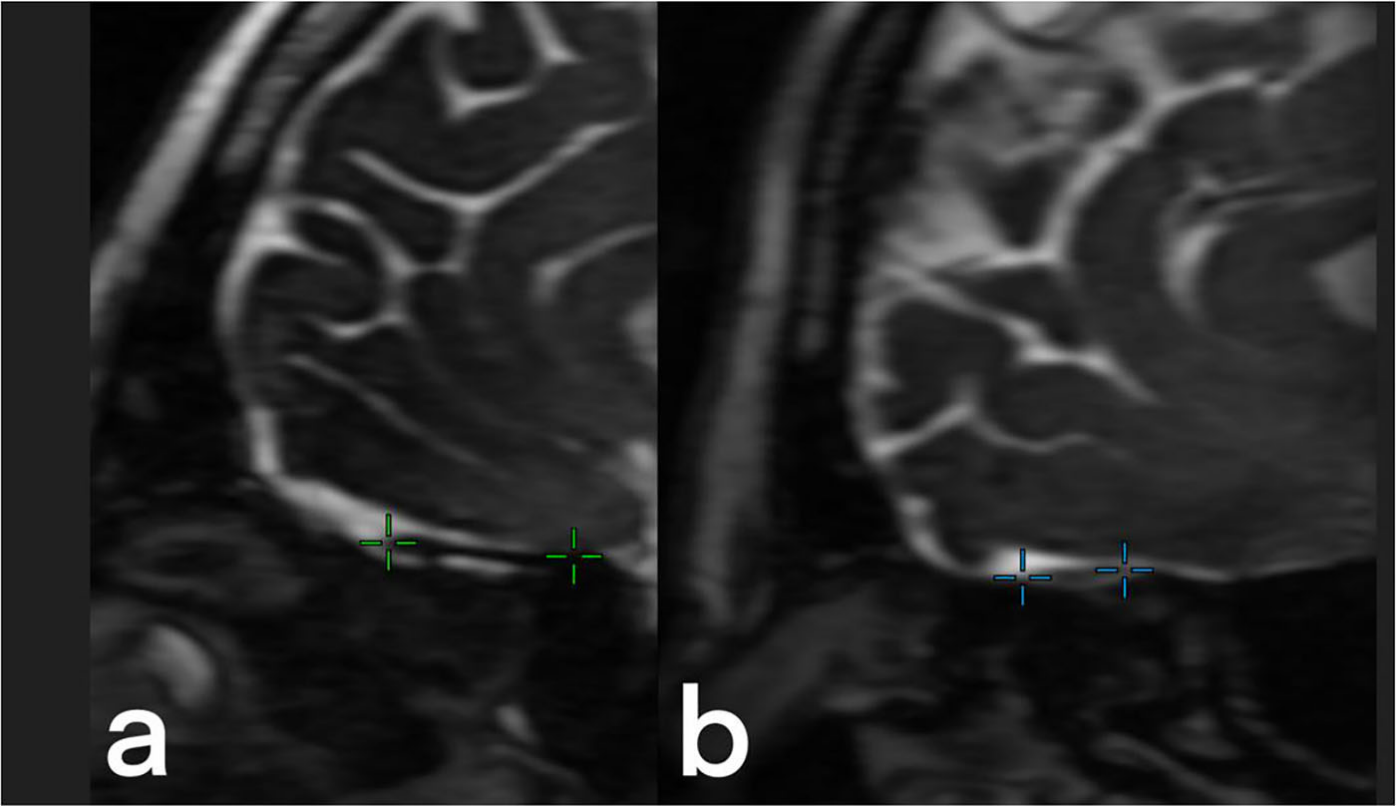
**MRI results of normal and abnormal olfactory tracts**. Sagittal multiplanar reconstruction of 3D‐FIESTA‐C MR images of (a) a 38‐year‐old male in the control group and (b) a 41‐year‐old male in the case group are seen to show normal and abnormal olfactory tracts (green and blue crosshairs, respectively).

Figure 5 presents the MRI imaging results of normal and abnormal olfactory tracts. While Figure 5a shows a healthy olfactory tract, Figure 5b indicates degeneration and an abnormal appearance.

## 4 Discussion

In this study, we examined the olfactory system structures of patients diagnosed with T2DM via MRI. The loss of sensation of smell is a highly significant and serious problem in T2DM patients. Over time, it may result in dejection, low quality of life, and low satisfaction with life. In addition to this, T2DM may experience changes in their eating habits due to the loss of this sensation and difficulty achieving normoglycemia (Le Floch et al. 1993). Despite this, it was reported that the loss of sensation of smell is mostly overlooked (Forbes and Cooper 2013). In a previous study, it was stated that glucose toxicity that could develop due to oxidative stress triggered the loss of sensation of smell (Gouveri et al. 2014). In a study that was performed with the participation of a pediatric sample at the ages of 3–17, it was found that epilepsy reduced OBV, but the type of epilepsy or the treatment method did not affect OBV, OTL, or OSD (Baykan et al. 2023). In a case–control study involving young adults with essential tremor, the OTL values of the control group were found to be significantly higher than those of the case group (Altunisik and Baykan 2019). Both of these studies (Altunisik and Baykan 2019; Baykan et al. 2023) emphasized that neurodegenerative diseases, similar to our results, could jeopardize the integrity of olfactory system structures. A study examined the effects of T2DM on the olfactory system using MRI. The aforementioned study found that patients with T2DM had lower cognitive and olfactory test scores than healthy individuals. Furthermore, it was reported that higher olfactory scores were associated with increased cortical thickness in the left parahippocampal gyrus and bilateral insula in T2DM. In the same study, it was emphasized that impaired olfaction in patients with T2DM could predict cognitive decline (Chen et al. 2022). The results of our study are similar to the literature.

In this study, there was a significant difference between the case and control groups in terms of their OBV, OTL, and OSD values according to MRI (*p* < 0.01). Previous studies have reported that impaired olfactory system structures in patients with T2DM may be a prognostic criterion for early diagnosis of cognitive impairment (Ramos‐Cazorla et al. 2024), increase the risk of Alzheimer's disease due to the resulting neurodegeneration (Reyes 2025), and may be associated with brain cortical thickness (Motaghi et al. 2024). In metabolic diseases including DM, the irregular secretion of insulin, leptin, orexins, neuropeptide Y, ghrelin, and cholecystokinin may lead to dysfunctions in the sensation of smell (Gouveri et al. 2014). In T2DM patients, loss of smell may indicate several disorders that have yet to show symptoms. As mentioned above, these may be summarized as irregular blood glucose levels, glucose toxicity, and the irregular secretion of several important molecules. In comparison to mortality, these are minor complications.

Cranial nerve III palsies in T2DM patients bring about peripheral neuropathy (Várkonyi et al. 2014). It is believed that diabetic neuropathy is associated with the sensation of smell. Its pathophysiology encompasses a set of processes related to cranial and central nervous system disorders (Várkonyi et al. 2014). The connection of olfactory dysfunction in T2DM with cranial nerve pairs and the central nervous system may make olfactory dysfunction/sensation loss a predictor of mortality. Previous studies have shown that there is a relationship between olfactory dysfunction and low OBV (Doğan et al. 2018; Haehner et al. 2008; Herzallah et al. 2013; Wang et al. 2011). In our study, the right, left, and total OBV values of the case group were significantly lower than those of the control group. The T2DM durations of the patients in the case group were negatively correlated with their OBV^R^, OBV^L^, OBV^T^, OTL^R^, and OTL^L^ values (*p* < 0.05). The HbA1c levels of the patients were positively correlated with their OTL^R^ and OSD^R^ values (*p* < 0.01).

Previous studies have demonstrated varying results on the relationship between olfactory disorders and neuropathy. Kaya et al. did not find a significant relationship between smell test classifications and the presence of nephropathy, microalbuminuria, or retinopathy (Kaya et al. 2020). Gouveri et al. and Weinstock et al. also did not observe a statistically significant relationship between DM and olfactory dysfunction (Gouveri et al. 2014; Weinstock et al. 1993). Rasmussen et al. compared 428 DM patients and 2776 healthy individuals and concluded that the prevalence of olfactory dysfunction in DM was higher (Rasmussen et al. 2018). The T2DM durations of the patients in the case group were negatively correlated with their OBV^R^, OBV^L^, OBV^T^, OTL^R^, and OTL^L^ values (*p* < 0.05). The T2DM durations of the patients in the case group was not correlated with their OSD^R^ (*p* > 0.05). The results of two previous studies on the relationship between T2DM duration and olfactory dysfunction varied (Le Floch et al. 1993; Naka et al. 2010).

The processes triggering olfactory dysfunction in T2DM patients are multidimensional. Some of these processes may be accompanied by nerve damage or neuropathy, while others may have developed independently of structural changes in the central nervous system. In a previous study, it was reported that smell exercises repaired olfactory dysfunction to a substantial extent when there was no accompanying neurological damage (Bulbuloglu and Altun 2021). The structural changes in the olfactory system structures of the T2DM patients in our study compared to the healthy controls suggested irreversible loss of smell. In addition to the need for further research, a previous study reported the protective role of metformin in the olfactory system (Assi et al. 2024). There is a need to investigate therapeutic interventions that will support olfactory recovery and preservation in future time. It was considered a limitation of this study that the case group was not followed up longitudinally. As this was a retrospective study, no smell test was carried out, and our results cannot be generalized to the entire population due to the small sample size.

## 5 Conclusion

The structural disorders of the olfactory system in patients with T2DM are affected by the duration of the disease and the elevation of HbA1c values. As the duration of T2DM increases, the increase in the structural disorders of the olfactory system necessitates blood glucose regulation, which suggests that these patients should adhere to diet and insulin treatment more strictly. In our study, compared to healthy controls, there was degeneration in the olfactory system structures of T2DM patients to varying degrees. This showed the reliability of MRI in the examination of olfactory structures. In addition to this, as it indicates olfactory nerve dysfunction and chemosensory involvement, smell disorders may be a predictor of serious conditions that could compromise the quality of life of T2DM patients and usually increase their mortality risk such as neuropathy and cranial nerve paralysis. In future studies, it is recommended to investigate therapeutic interventions that can protect olfactory system structures in T2DM.

## Author Contributions

**Aski Vural**: writing – original draft, funding acquisition, data curation, conceptualization, methodology, investigation. **Erman Altunişik**: project administration, methodology, conceptualization, formal analysis, data curation, project administration, resources. **Suat Kamil Sut**: project administration, software, conceptualization. **Sukru Sahin**: writing – review and editing, conceptualization, methodology, validation, supervision, visualization. **Ali Haydar Baykan**: project administration, software, conceptualization.

## Funding

The authors have nothing to report.

## Conflicts of Interest

The authors declare no conflicts of interest.

## Data Availability

The data that support the findings of this study are available on request from the corresponding author. The data are not publicly available due to privacy or ethical restrictions.
